# Primary aim results of a clustered SMART for developing a school-level, adaptive implementation strategy to support CBT delivery at high schools in Michigan

**DOI:** 10.1186/s13012-022-01211-w

**Published:** 2022-07-08

**Authors:** Shawna N. Smith, Daniel Almirall, Seo Youn Choi, Elizabeth Koschmann, Amy Rusch, Emily Bilek, Annalise Lane, James L. Abelson, Daniel Eisenberg, Joseph A. Himle, Kate D. Fitzgerald, Celeste Liebrecht, Amy M. Kilbourne

**Affiliations:** 1 Department of Health Management and Policy, School of Public Health, University of Michigan, SPH II, 1415 Washington Heights, Ann Arbor, MI 48109 USA; 2 Department of Psychiatry, Michigan Medicine, University of Michigan, Ann Arbor, USA; 3 Survey Research Center, Institute of Social Research, University of Michigan, Ann Arbor, USA; 4 Department of Statistics, University of Michigan, Ann Arbor, USA; 5 Department of Health Policy and Management, UCLA, Los Angeles, USA; 6 School of Social Work, University of Michigan, Ann Arbor, USA; 7 Department of Psychiatry, Columbia University Irving Medical Center/New York State Psychiatric Institute, New York City, USA; 8 Department of Learning Health Sciences, Michigan Medicine, University of Michigan, Ann Arbor, USA; 9 Quality Enhancement Research Initiative (QUERI), US Department of Veterans Affairs, Washington, D.C., USA

**Keywords:** Mental health, Schools, Cognitive behavioral therapy, Facilitation, Adaptive implementation strategies, Coaching, Adolescent mental health

## Abstract

**Background:**

Schools increasingly provide mental health services to students, but often lack access to implementation strategies to support school-based (and school professional [SP]) delivery of evidence-based practices. Given substantial heterogeneity in implementation barriers across schools, development of *adaptive implementation strategies* that guide which implementation strategies to provide to which schools and when may be necessary to support scale-up.

**Methods:**

A clustered, sequential, multiple-assignment randomized trial (SMART) of high schools across Michigan was used to inform the development of a school-level adaptive implementation strategy for supporting SP-delivered cognitive behavioral therapy (CBT). All schools were first provided with implementation support informed by Replicating Effective Programs (REP) and then were randomized to add in-person Coaching or not (phase 1). After 8 weeks, schools were assessed for response based on SP-reported frequency of CBT delivered to students and/or barriers reported. Responder schools continued with phase 1 implementation strategies. Slower-responder schools (not providing ≥ 3 CBT components to ≥10 students or >2 organizational barriers identified) were re-randomized to add Facilitation to current support or not (phase 2). The primary aim hypothesis was that SPs at schools receiving the REP + Coaching + Facilitation adaptive implementation strategy would deliver more CBT sessions than SPs at schools receiving REP alone. Secondary aims compared four implementation strategies (Coaching vs no Coaching × Facilitation vs no Facilitation) on CBT sessions delivered, including by type (group, brief and full individual). Analyses used a marginal, weighted least squares approach developed for clustered SMARTs.

**Results:**

SPs (*n* = 169) at 94 high schools entered the study. *N* = 83 schools (88%) were slower-responders after phase 1. Contrary to the primary aim hypothesis, there was no evidence of a significant difference in CBT sessions delivered between REP + Coaching + Facilitation and REP alone (111.4 vs. 121.1 average total CBT sessions; *p* = 0.63). In secondary analyses, the adaptive strategy that offered REP + Facilitation resulted in the highest average CBT delivery (154.1 sessions) and the non-adaptive strategy offering REP + Coaching the lowest (94.5 sessions).

**Conclusions:**

The most effective strategy in terms of average SP-reported CBT delivery is the adaptive implementation strategy that (i) begins with REP, (ii) augments with Facilitation for slower-responder schools (schools where SPs identified organizational barriers or struggled to deliver CBT), and (iii) stays the course with REP for responder schools.

**Trial registration:**

ClinicalTrials.gov, NCT03541317, May 30, 2018.

**Supplementary Information:**

The online version contains supplementary material available at 10.1186/s13012-022-01211-w.

Contributions to the literature

- Schools are promising venues for students accessing evidence-based mental health services, but the development of *adaptive implementation strategies* to support schools and school professionals may be necessary for scale-up.
- The effectiveness of different implementation strategies for supporting SP-delivered cognitive behavioral therapy was examined in Michigan high schools.
- Among four compared strategies, the most effective strategy in terms of average CBT delivery by SPs is the *adaptive implementation strategy* that begins with Replicating Effective Program (REP; a low-burden, low-cost strategy), augments with Facilitation for slower-responder schools (schools where SPs identified organizational barriers or struggled to deliver CBT), and continues with REP for responder schools.

## Background

Depression and anxiety disorders impact approximately 15% and 30% of school-aged youth, respectively [1], and are increasing. Several evidence-based practices (EBPs), including cognitive behavioral therapy (CBT), can improve clinical outcomes for adolescents [2–6], but barriers to care limit access, with less than one in five teens receiving any kind of evidence-based care [7]. Barriers include cost, stigma, and a limited number of behavioral health providers, particularly in rural communities [8–13]. More than half of mental disorders begin during school-age years [14]. Untreated, these illnesses can impair development and academic performance and lead to poor physical and mental health outcomes, including suicide, self-injury, and substance abuse [2, 15–18] at substantial social and economic cost [2, 15, 19].

Given barriers to community-based care, schools have increasingly served as *de facto* providers of mental health care services. Schools provide a low stigma, convenient, and sustainable setting to overcome treatment barriers. Youth spend a great deal of time in school, and most have daily access to school professionals (SPs; social workers, counselors, psychologists) who can provide mental health and substance use support at no additional cost to families, in a familiar environment [19–21]. Students are also more willing to access mental health services in school than community settings [11, 12]. Between 2012 and 2015, nearly 60% of students receiving mental health services reported receiving some in school, and nearly 40% reported receiving services in schools exclusively [22]. Students receiving care exclusively in schools were disproportionately lower income, underrepresented minorities, and/or on public insurance [22].

Schools, however, face their own barriers to providing effective mental health care. SPs rarely have access to training in mental health EBPs, such as CBT, or the support they need to provide EBPs sustainably [23] and have reported low confidence in their ability to deliver treatments like CBT [24–26]. Organizational barriers, including competing demands on SP time, lack of (or barriers to accessing) space or other school resources, and lack of support by school or district administrators [27], also impede SP ability to provide CBT or other EBPs in schools.

Implementation strategies—or theory-based techniques “used to enhance the adoption, implementation, or sustainability of an [EBP]” [28, 29]—hold potential for improving SP delivery of EBPs like CBT in schools. Replicating Effective Programs (REP), Coaching, and Facilitation are three promising school-level implementation strategies that have the potential to mitigate barriers to SP-delivered CBT. REP, detailed below, is a relatively low-burden, low-cost, readily scalable strategy that packages EBP training with on-demand technical assistance (TA) to customize the EBP to local users’ (e.g., schools’) needs [30, 31]. REP addresses fundamental barriers to school-based EBP delivery and has been shown to improve the uptake of psychosocial EBPs in community-based settings across different community organizations and health systems [31–35]. However, REP’s low intensity may prove inadequate for schools where SPs require substantial skills training or where organizational barriers are significant [35, 36]. As such, some schools may require REP augmentations that provide more intensive support. For skills-related barriers, a Coaching model that provides SPs with more intensive post-training support through skills modeling, practice, and feedback has shown promise for promoting EBP delivery [24, 26, 37, 38]. For organizational barriers, Facilitation—based on the integrated-Promoting Action on Research Implementation in Health Services (i-PARIHS) [39] framework—provides schools with ongoing consultation from an expert in strategic thinking and EBP implementation to garner administrative support, solve logistical challenges, and build community buy-in. In several community-based cluster-randomized trials, Facilitation has been shown to improve mental health EBP uptake [30, 34, 40–45] and to be highly cost-effective [46, 47].

**Fig. 1:**
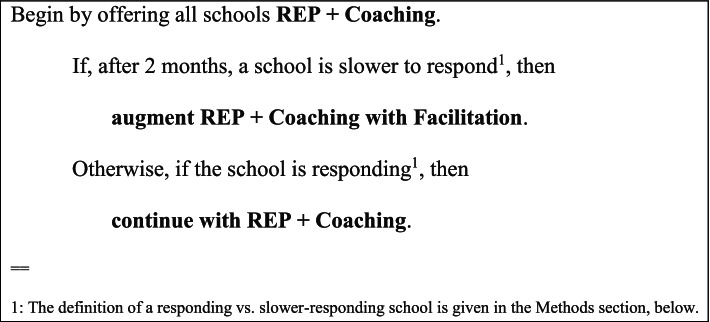
Example of a higher-intensity adaptive implementation strategy

Given that there is substantial heterogeneity in terms of implementation barriers at schools [48] and in how different schools might respond to different combinations of strategies, there is a need to develop and evaluate effective *adaptive implementation strategies* [49–51]. An adaptive implementation strategy is a sequence of decision rules used to guide implementers in selecting which combination of implementation strategies (e.g., REP, Coaching, Facilitation) to offer and when, including considerations of a school’s changing needs. An example of a higher-intensity adaptive implementation strategy is shown in Fig. 1.

However, there is currently no research that evaluates the effectiveness of this type of adaptive implementation strategy for improving CBT delivery in schools. Perhaps more fundamentally, there is no research to inform (i) the effectiveness of starting with REP vs. REP + Coaching, (ii) the effectiveness of augmenting with Facilitation vs. not among schools that are slower to respond to REP or REP + Coaching, or (iii) whether additional school factors ought to be taken into consideration when making decisions, e.g., to start with REP vs. REP + Coaching, or to augment with Facilitation vs. not among slower-responder schools.

The current study (R01MH114203)—The Adaptive School-based Implementation of CBT (ASIC) Study [51]—used a clustered, sequential, multiple-assignment randomized trial (SMART) [52] to inform the development of a school-level adaptive implementation strategy for adopting and scaling up SP delivery of CBT. ASIC was done in partnership with TRAILS (Transforming Research into Action to Improve the Lives of Students) [26], a program that aims to implement CBT in high schools across the state of Michigan.

This manuscript reports the results of ASIC’s primary research aim, which was to evaluate the effectiveness of the adaptive implementation strategy shown in Fig. 1 versus providing REP alone (not adaptive, no Coaching, no Facilitation). The primary outcome is the *total number* of SP-reported CBT sessions delivered to students by SPs over the 18-month study period. Secondary outcomes are the number of CBT sessions delivered by *type of session*: group vs. individual brief vs. individual full. We hypothesized that, compared to REP alone, the adaptive implementation strategy in Fig. 1 would lead to a higher total number of CBT sessions delivered, on average, over 18 months.

We also present outcomes for two other implementation strategies embedded within ASIC: REP + Coaching from the start for all schools (not adaptive, no Facilitation), and REP that is augmented with Facilitation for slower-responder schools and continued REP for responder schools (REP + Facilitation; adaptive, no Coaching).

## Methods

### Participants and eligibility

SPs at eligible Michigan high schools were recruited for study participation.

Schools were eligible to participate in ASIC if they:

1. Served high school students (grades 9–12) from a Michigan school district and had not previously participated in a school-based CBT implementation initiative
2. Were within a 2-h driving distance of a mental health professional who was trained by TRAILS and able to serve as one of the Coaches for the implementation study
3. Had at least one eligible SP that agreed to participate in study assessments for the duration of the study
4. Had minimally sufficient resources, including space to deliver CBT, to allow for delivery of individual and/or group mental health support services on school grounds but outside of the general classroom environment

Eligible SPs were:

1. Employed at an eligible Michigan high school
2. Had a background in clinical school social work, counseling, psychology, or similar field, and were able to meet with students regularly to deliver mental health support services outside of the general classroom environment
3. Able to read and understand English, comprehend study assessments, and give informed consent
4. Completed a 1-day didactic training in CBT

Recruitment of schools was done by first contacting SPs at schools and then contacting school administrators. Specifically, once SPs confirmed interest and eligibility, a principal or other senior school administrator was asked to provide data on building-wide socio-demographics and leadership priorities regarding EBPs. While SPs may sometimes work in multiple schools, in this study, all SPs were associated with only one ASIC-enrolled school.

### Evidence-based program to be implemented: cognitive behavioral therapy (CBT)

This study focused on encouraging SP delivery of CBT for students with depression and/or anxiety. Modular CBT, wherein individual CBT components can be delivered flexibly and responsively depending on student needs, was selected given its strong evidence base [53, 54]. Modular CBT has been found to reduce symptoms of depression and anxiety relative to usual care [54–58], including with school-age youth [57, 59] and across different racial or ethnic groups [9, 56]. Furthermore, CBT has demonstrated effectiveness when delivered within school settings [60–62]. CBT components included psychoeducation, relaxation, mindfulness, cognitive restructuring, behavioral activation, and exposure and were defined based on established, evidence-based intervention protocols [63, 64] and an established “distillation” model [65].

### Implementer: the TRAILS program

TRAILS (not research staff) coordinated and delivered all implementation strategies. Specifically, TRAILS delivered the in-person, didactic CBT trainings and REP TA; recruited and trained all Coaches [26, 66]; recruited and trained the Facilitator; delivered phase 1 Coaching; monitored schools for improvement (e.g., determined responder status at the end of phase 1); and delivered phase 2 strategies (including Facilitation).

### Clustered, sequentially randomized trial design

**Fig. 2:**
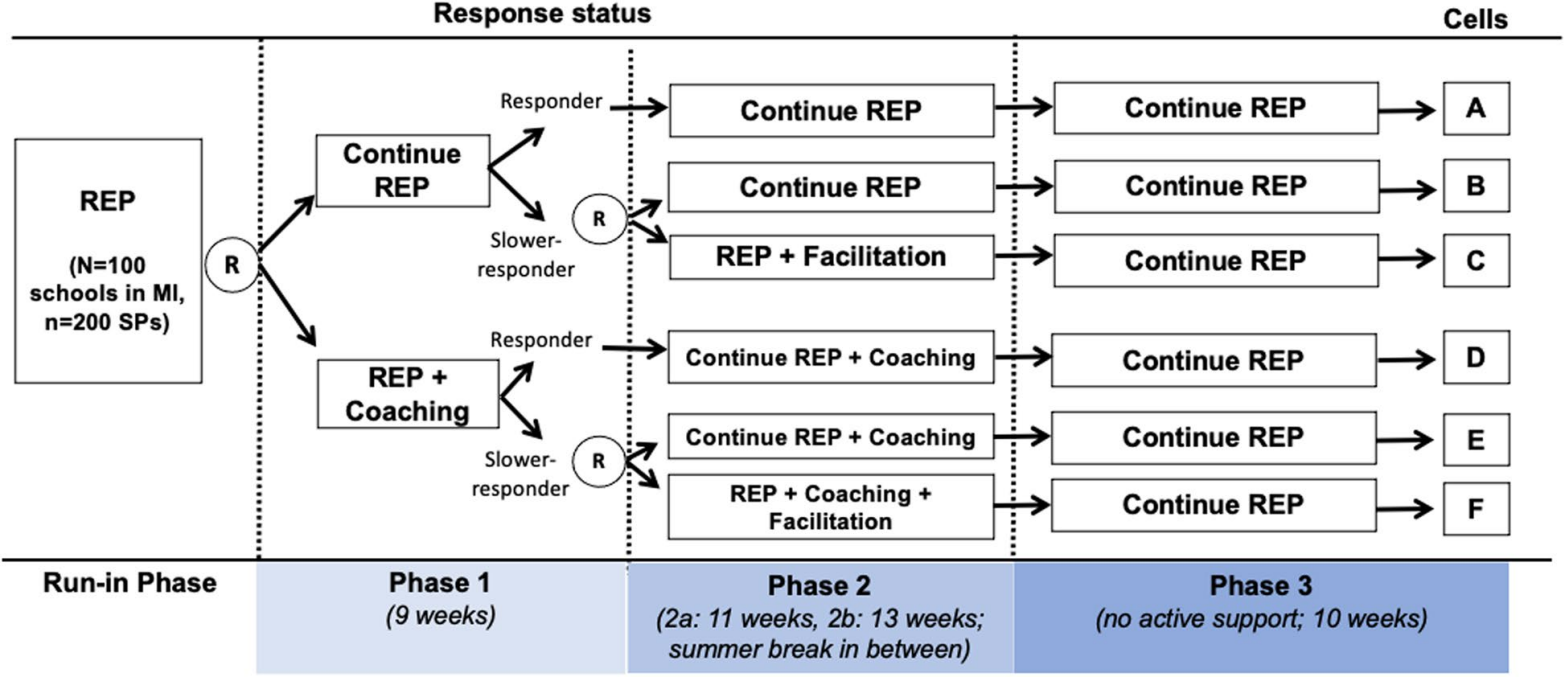
Full ASIC trial design

ASIC used a four-phase, clustered SMART [52] (Fig. 2). The study spanned four phases (9–13 weeks each) across two school years. Full ASIC study details—including rationale, stratified randomizations, pre-specified primary outcome, and sample size calculations—are available elsewhere [51].

#### Run-in phase (pre-randomization)

The pre-randomization, run-in phase began in October 2018. All schools were offered REP for up to 3 months. SPs were provided with information on registering for the study data collection dashboard (see below). Two weeks prior to the first randomization (mid-January 2019), as part of REP, TRAILS offered SPs a 1-day didactic training in CBT.

#### Phase 1

Approximately 2 weeks following training (late January 2019), schools were randomized with equal probability to either augment REP with Coaching or not, marking the beginning of phase 1.

#### Phases 2a and 2b

In early April 2019, 8 weeks after the first randomization, schools identified by TRAILS as “slower responders” (defined below) were re-randomized with equal probability to augment with Facilitation or not. Phase 2 spanned two semesters, separated by the summer break. We label these phases 2a (remainder of Spring 2019 semester) and 2b (Fall 2019 semester).

#### Phase 3

At the end of the Fall 2019 semester (December 2019), Coaching and Facilitation were discontinued and all schools completed the study with access only to REP supports. Data collection continued through mid-April 2020.

### Implementation strategies

#### Replicating Effective Programs

All schools were offered REP [32]. REP, based on Rogers’ diffusion model [67] and social learning theory [68], is a low-intensity strategy designed to enhance EBP uptake through development of customized intervention materials appropriate for the specific implementation setting, didactic training, and provision of low-level TA. Prior to ASIC, TRAILS developed customized CBT materials designed to address common symptoms of depression and anxiety, tailored for school-based delivery by SPs. All materials were made available to SPs through the TRAILS website (http://www.trailstowellness.org). Materials included standardized screening tools SPs could use to identify students appropriate for CBT, an overview of CBT components, session agendas for providing CBT to individual students or groups, talking points for teaching students about CBT, and CBT handouts, worksheets, and resources for use with students. TA to support SP delivery was provided by a PhD-level clinical psychologist board-certified in CBT and included a monthly newsletter with information on TRAILS resources, a monthly opt-in TA call, and contact information for as-needed support via phone or email.

REP also included a daylong, in-person didactic training. TRAILS staff clinicians (PhD- and LMSW-level practitioners) offered the training at several locations across Michigan. The training covered screening and identification of students, CBT core components, and theoretical underpinnings. Training strategies included didactic instruction, videos and live demonstrations, role-plays with feedback, and facilitated small group discussion.

#### Responder vs. slower-responder schools

REP included a monitoring strategy whereby, at the end of phase 1, TRAILS identified whether schools were responders or slower-responders through a short assessment to all SPs (Supplementary Appendix A). School-level responder/slower-responder status was determined for the explicit purpose of making the decision to offer Facilitation or not. (Recall that Facilitation is a school-level strategy intended to impact school-level processes and barriers.) Schools were categorized as “slower-responders” if any SPs reported not providing ≥3 CBT components to ≥10 students *OR* if SPs reported, on average, >2 barriers to CBT delivery. Slower-responder schools thus included (i) schools where any SPs struggled to deliver CBT and (ii) schools where SPs were delivering CBT but endorsed barriers potentially precluding long-term delivery or sustainability. Finally, (iii) schools where any SPs failed to complete the monitoring assessment were also considered slower responders.

The responder/slower-responder definition was based on pilot data with non-ASIC SPs that found that SPs tended to report no barriers or 3+ barriers, and those reporting barriers had poor prognosis for implementation without access to a strategy (like Facilitation) designed to address these barriers. From this pilot data, schools identified as “responders” were not thought to be in need of Facilitation; schools identified as “slower-responders” were thought to *potentially* benefit from Facilitation.

#### Coaching

The Coaching implementation strategy, used by TRAILS in more than 20 Michigan high schools prior to ASIC [26], was derived from the school-based Positive Behavior Interventions and Supports (PBIS) model of coaching for individual development [69]. A comprehensive, operationalized coaching protocol guides TRAILS Coaches to support SP learning and CBT delivery. In addition, the Coaches were expected to attend ~12 SP-delivered CBT student group sessions, during which Coaches would observe SP CBT delivery, provide feedback [70, 71], and, as appropriate, model the use of CBT components to improve SP competence [69, 72–76]. Each school was assigned a Coach with whom they were to arrange weekly Coaching visits for a minimum of one semester. TRAILS then administered a short, objective CBT competency assessment to all SPs; Coaches completed standardized ratings of their assigned SPs. Schools were provided with either a second semester of in-person Coaching (when SPs showed gaps in competency) or a stepped-down version (when SPs demonstrated sufficient competency). Coaches were required to complete weekly logs, documenting interaction with their assigned SPs and utilization of specific coaching techniques.

Coaches were recruited and trained by TRAILS. Coaches were typically licensed community mental health (CMH) providers (e.g., Licensed Clinical Social Workers) serving in child- or family-treatment roles and were recruited through professional networking or contacts made to CMH clinical directors. To be eligible to serve as a Coach for ASIC, Coaches had to complete an initial didactic training in CBT and mindfulness, 15 weeks of one-to-one consultation with a TRAILS staff clinician, and a second didactic training focused on the TRAILS Coaching Protocol [66].

#### Facilitation

Facilitation is based on the i-PARIHS Framework [77] and was designed to improve CBT delivery by improving SP self-efficacy [78] through mitigation of organizational (i.e., school-level) barriers. All SPs at schools assigned to Facilitation had the opportunity to engage in regular phone calls with the Facilitator for up to 24 weeks (the duration of phase 2). In line with prior studies [34, 40, 41, 79], the Facilitator addressed local barriers to CBT delivery by supporting SPs in the development of strategic thinking, leadership skills, and amelioration of barriers to CBT delivery through a five-step process. This process includes helping SPs set measurable goals, aligning SP strengths and CBT delivery with existing school and administrator values and priorities, providing guidance on overcoming local barriers to CBT delivery, engagement with administrators and other key stakeholders, and communication and marketing regarding the added value of CBT delivery (Table 1) [34, 41, 77, 80, 81]. To encourage positive synergy at schools that previously had been offered Coaching, the Facilitator could encourage SPs to discuss CBT skill-development issues and/or discuss strategies for improving communication with their Coach.

**Table 1:** Five-step Facilitation process

***Main focus and description of key activities for each Facilitation step***	
(1) **Initiation and benchmarking** to better understand barriers and set goals. *The Facilitator contacts SPs to give background on CBT research and evidence; discusses common barriers to using CBT (e.g., administrator support, protected time), and works with SPs to begin setting measurable goals for CBT uptake.*	
(2) **Mentoring SPs** through regularly scheduled calls designed to rally motivation and encourage strategic thinking. *The SPs and the Facilitator hold regular calls (suggested weekly) to help develop rapport; discuss and prioritize anticipated and experienced barriers and facilitators to CBT delivery; the Facilitator provides SPs with guidance for overcoming specific barriers to CBT uptake (e.g., facilitating communication with school administrator, parents, or other stakeholder groups). As necessary, the Facilitator connects SPs with REP TA or (if appropriate) their assigned Coach.*	
(3) **Developing an action plan** to mitigate or overcome barriers to adoption of CBT use. *The Facilitator works with SPs to design a plan and timeframe for addressing specific barriers, including establishing and tracking key metrics for success (e.g., CBT delivery).*	
(4) **Leveraging influence** by assisting SPs in discerning school, community, and administrative priorities, and encouraging SPs to communicate to stakeholders how CBT aligns with broader priorities. *The Facilitator continues to work with SPs and also reaches out to administrators or other leaders to help identify school/community priorities, and help SPs align CBT use and goals with these existing values and priorities. The Facilitator also works with SPs to describe how CBT aligns with leadership priorities and adds value for students, administrators, and other school employees (e.g., instructional staff).*	
(5) **Ongoing marketing**, wherein the Facilitator summarizes progress and develops plans for sustaining program delivery. *The Facilitator helps SPs summarize achievements, progress, continued barriers, and alignment with other school priorities or initiatives, and also helps to develop sustainability plans (e.g., by showcasing CBT's added value).*	

Facilitation was provided by a PhD-level clinical psychologist with expertise in CBT delivery, strategic thinking, and school-based mental health delivery. The Facilitator received training in Facilitation through the Quality Enhancement Research Initiative (QUERI) for Team-based Behavioral Health [82].

### Primary and secondary aims and hypotheses

The study includes four embedded implementation strategies (Table 2), two of which were adaptive based on response to phase 1 strategies [49–51]. The primary aim was to test whether the least intensive strategy—REP alone—versus the adaptive implementation strategy in Fig. 1—REP + Coaching + Facilitation—results in a difference in terms of average total CBT delivery (shaded rows in Table 2). We hypothesized that, on average, REP + Coaching + Facilitation would lead to greater CBT delivery than REP alone.

**Table 2:**
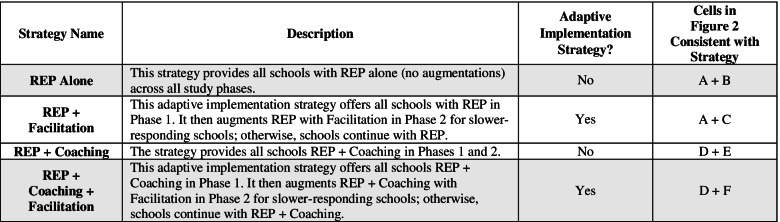
Four embedded implementation strategies

Shaded cells indicate the two strategies compared for primary aim analysis

As a secondary aim, we present results for all other pairwise comparisons for primary outcome and secondary outcomes. For all outcomes, we hypothesized that the four strategies would be ordered as follows, from greater to lesser amount of average CBT delivery:

- REP + Coaching + Facilitation > REP + Coaching = REP + Facilitation > REP Alone.

### Research measures

#### Quarterly surveys

SPs completed baseline and quarterly research surveys that included demographics, professional qualifications and duties, prior training in and exposure to CBT, CBT knowledge and comfort with delivery, and barriers to delivery. SPs received $10 for each survey completed. SP demographics are reported in Supplementary Appendix B.

#### Outcomes: CBT delivery

**Fig. 3:**
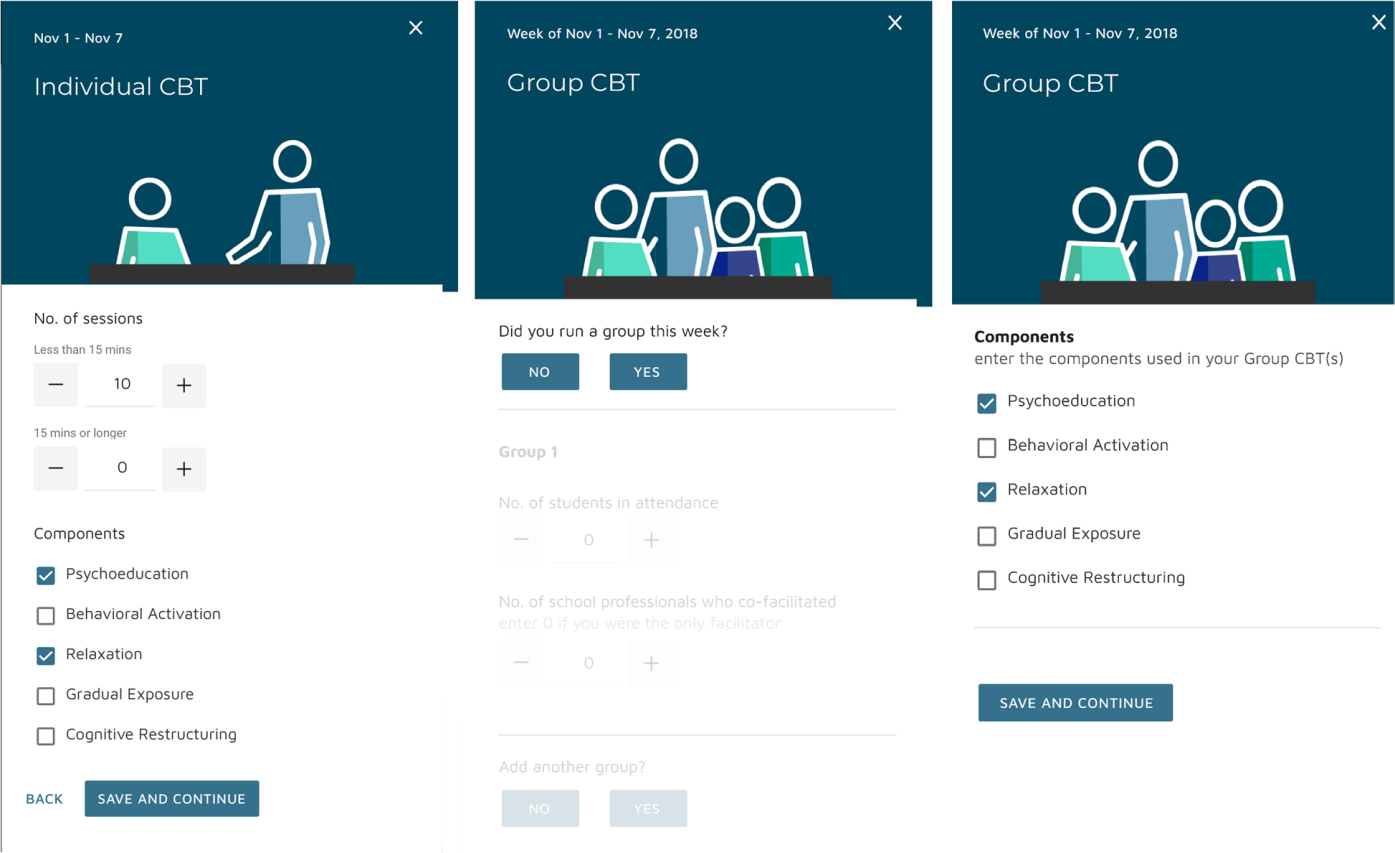
Individual and group CBT reporting on the ASIC dashboard

The primary outcome was the total number of self-reported CBT sessions delivered by SPs (hereafter: *CBT delivery*). SPs were asked to self-report their CBT delivery weekly through a secure dashboard used explicitly for research purposes (Fig. 3). Each weekly report included the number of group sessions and full (≥15 min) and brief (<15 min) individual sessions delivered. To minimize burden, SPs were also provided with physical weekly tracking notepads and could enter dashboard data for up to 4 weeks retrospectively. SPs received $3 for each weekly report provided and were encouraged to report even if/when they delivered no CBT.

The primary outcome was the total number of CBT sessions delivered (group + brief individual + full individual), and three secondary measures were total CBT delivery by type: group, full, and brief. Weekly CBT delivery data collection took place phases 1 through 3, except during summer break or known school holidays (e.g., winter holidays).

#### Impact of COVID-19 on research data collection

Data collection was planned through mid-April 2020 (60 weeks total). However, due to the COVID-19 pandemic, Michigan closed schools statewide starting March 16, 2020 (week 56 of study data collection) [83, 84]. Thus, analyses included data through week 55.

### Analyses

To analyze the data, we used a generalization of a marginal, weighted least squares approach specifically developed to ensure unbiased estimation of the comparison of the implementation strategies embedded in a clustered SMART. The method is a generalization of the approach described in [52] for accommodating a repeated measures outcome (total CBT delivery by phase).

#### Study sample

In accordance with intent-to-treat principles, all *n =* 169 SPs at all *N =* 94 schools randomized in phase 1 were included in all analyses.

#### Modeling and estimation

The same modeling and estimation strategy was used for the primary outcome (average total CBT delivery) and for each of the three secondary outcomes (average total CBT delivery by type).

At each phase (1, 2a, 2b, and 3), a separate regression model was fit for the CBT delivery outcome, as follows: The phase 1 regression included an intercept and a contrast coded (+1/−1) indicator for phase 1 strategy. The phase 2a, 2b, and 3 regression models included an intercept, a contrast-coded indicator for phase 1 strategy, a contrast-coded indicator for phase 2 strategy, and the interaction between phase 1 and phase 2 strategies. All models included the following pre-specified, school-level baseline covariates: school size (>500 or ≤ 500 students), location of school (rural or urban), percentage of students on free/reduced lunch program (≥ 50% or <50%), and pre-randomization CBT delivery (any vs. none), as well as school-aggregated SP education and job tenure.

Phase 1 outcomes cannot be impacted by strategies offered in the future (i.e., in phase 2), whereas phase 2a, 2b, and 3 outcomes can be impacted by the sequence of strategies offered in phases 1 and 2; the fitted regression models reflect this feature of the SMART [85].

Standard least squares was used to estimate the phase 1 regression model. Weighted and replicated generalized estimating equations were used to estimate the regression models in phases 2a, 2b, and 3 [52]. As slower-responder schools were randomized twice with probability 1/2, they had a 1/4 chance of following their assigned sequence of strategies, whereas responder schools, randomized once with probability 1/2, had a 1/2 chance of following their assigned strategy sequence. Weighting is used to account for this known under-representation of slower-responder schools. Specifically, data for SPs in slower-responder schools were assigned a weight of 4, whereas data for SPs in responder schools were assigned a weight of 2. In addition, since each group of responder schools is consistent with two strategies (i.e., schools in cell A are consistent with REP only and REP + Facilitation and schools in cell D with REP + Coaching and REP + Coaching + Facilitation; see Table 2), the data for these schools was used twice (i.e., replicated) to facilitate a more efficient comparison of the four strategies. For details, see [52].

All models used bootstrapped standard errors (based on 1000 samples) to account for (i) clustering of SPs within schools, (ii) multiple observations per SP, (iii) sampling variation in the unknown distribution of the weights, and (iv) replication.

The fitted regression models were used to calculate estimates of the average CBT delivery at each phase, under each of the four strategies. To facilitate the comparison of the strategies using a single-number summary, for each of the four strategies, the phase-specific averages were summed to calculate “average total CBT delivery.” As phases varied slightly in length, in secondary analyses, we also computed average weekly delivery during each phase by dividing average delivery during each phase by the number of phase-weeks (results are provided in Supplementary Appendix C).

#### Primary aim comparison

ASIC’s primary aim was to test the null hypothesis that there is no difference in average total CBT delivery (primary outcome) between the least intensive strategy—REP alone—and the adaptive implementation strategy in Fig. 1—REP + Coaching + Facilitation. A Wald test, calculated as the pairwise comparison divided by its estimated standard error, was used to test this null hypothesis.

#### Secondary aim comparisons

For each outcome, all pair-wise comparisons (and associated 95% confidence intervals) of the average total CBT sessions delivered were estimated to better understand how the four strategies compared to each other.

#### Effect sizes

To enhance clinical interpretation, effect sizes [86] were calculated for each pairwise difference. Effect sizes were calculated as the pairwise comparison divided by an estimate of the standard deviation of the average total CBT delivery. Effect sizes of 0.2, 0.5, and 0.8 were regarded as small, moderate, and large, respectively [86].

#### Missing data

Multiple imputation was used to replace missing values in the outcomes and other measures [87]. Forty data sets were generated. All estimates, standard errors, and hypothesis tests reported below were calculated using standard rules [88, 89] for combining the results of identical analyses performed on each of the 40 imputed data sets. All regression models were fit with and without multiply-imputed data and results did not change substantively (details in Supplementary Appendix D).

## Results

### Participants and baseline data

Michigan schools (*N* = 312) were approached for participation, with *N* = 115 (*n* = 227 SPs) agreeing to participate. One hundred sixty-nine SPs at *N* = 94 schools completed training and were randomized. The most common SP roles (*n* = 169) were school counselor (59%) and social worker (23%); other roles (18%) included school psychologist, behavioral intervention specialist, and special education teacher. SPs had been in their roles for an average of 8 years (standard deviation (SD) = 7.7) and 153 (90.5%) reported some graduate education. Twenty-one percent (*n* = 35) served exclusively or primarily students in special education; the remainder served students in general education or both. Ninety-two percent (*n* = 156) reported seeing students for individual counseling and 58 (34%) reported convening student groups. Fifty-seven SPs (34%) reported prior formal training in CBT (e.g., lectures in a graduate course) and 77 (46%) informal training (e.g., brief presentation, self-directed readings) (Supplementary Appendix B). Sixty-one percent (*n* = 104) were at schools where at least one SP reported delivering CBT during the pre-training phase.

**Fig. 4:**
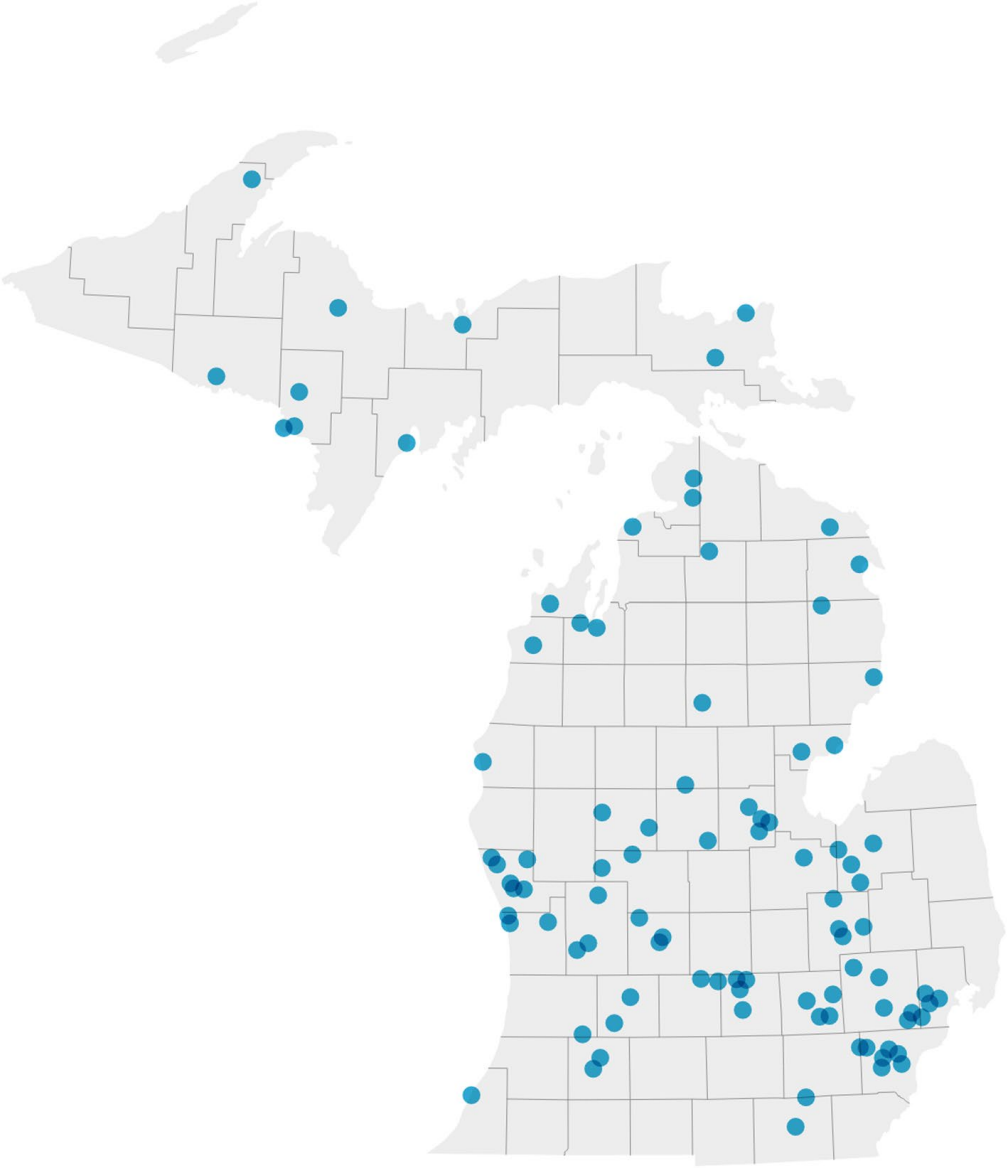
Map of Michigan High Schools enrolled in ASIC. Note: *N* = 94 schools participated. School location on the map was determined by the school address listed on the school’s website

Figure 4 shows the *N* = 94 ASIC schools within Michigan. Fifty-six percent were rural; average school size was 869 students (SD = 600) with 44% (SD = 18) qualifying for free/reduced lunch. Most schools had either 1 (*N* = 38; 40%) or 2 (*N* = 37; 39%) participating SPs.

### Strategies assigned and received

**Fig. 5:**
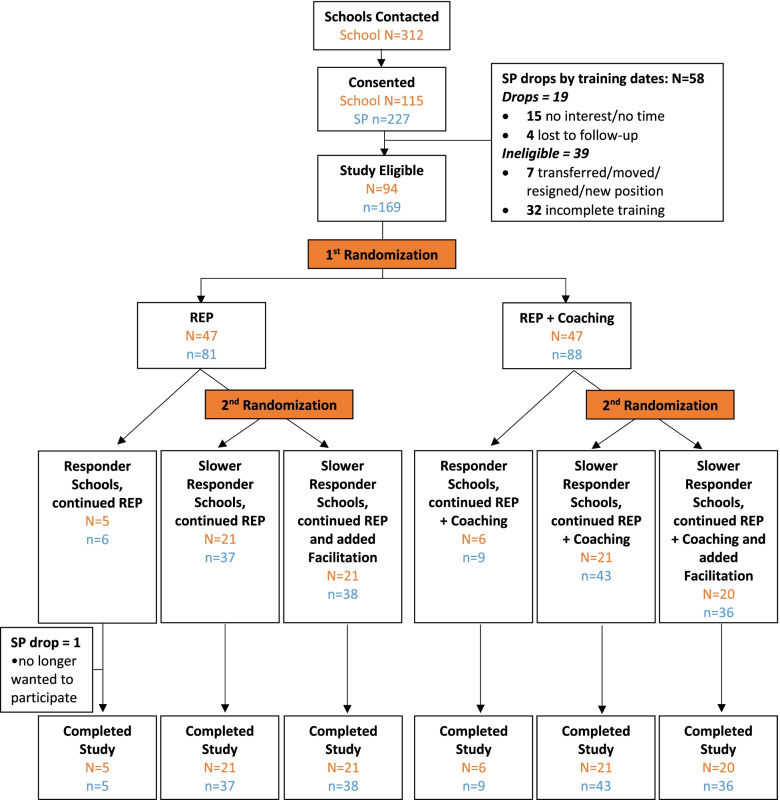
CONSORT diagram for the ASIC study — schools and school professionals

In phase 1, schools were randomized to Coaching (*n* = 88 SPs in *N* = 47 schools) vs. no Coaching (*n* = 81 SPs in *N* = 47 schools; Table 3). *N* = 33 schools (70%) assigned to Coaching were documented as ever engaging in Coaching. At the end of phase 1, 83 schools (88%, 154 SPs) were deemed slower-responders and re-randomized. *N* = 41 slower-responder schools (*n* = 74 SPs) were re-randomized to augment with Facilitation (Table 4; Fig. 5). *N* = 41 schools (100%) were documented as ever engaging in Facilitation. TA was provided to all SPs under REP; however, engagement was minimal, with few SPs attending monthly opt-in calls and 270 total minutes of TA on-demand support documented across the entire study period.

**Table 3:** School-level characteristics by phase 1 randomization (Coaching vs. no Coaching) (*N* = 94 schools)

	All schools (***N*** = 94)	REP (***N*** = 47)	REP + Coaching (***N*** = 47)
**Proportion of schools with >500 students (vs. ≤ 500 students)**	0.65	0.66	0.64
**Proportion of rural schools (vs. non-rural)**	0.56	0.55	0.57
**Proportion of schools with >50% students on free/reduced lunch program (vs. ≤ 50%)**	0.36	0.36	0.36
**Proportion of schools with any pre-randomization CBT delivery (vs. none)**	0.57	0.55	0.60
**School-level average SP tenure in years: mean (SD)**	7.56 (5.96)	7.34 (6.03)	7.79 (5.94)
**School-level proportion of SPs with graduate degree: mean (SD)**	0.90 (.26)	0.89 (.28)	0.91 (.24)
**Number of SPs per school**	Mean = 1.80	Mean = 1.72	Mean = 1.87
	1 SP: *N* = 38	1 SP: *N* = 21	1 SP: *N* = 17
	2 SPs: *N* = 37	2 SPs: *N*= 18	2 SPs: *N* = 19
	3 SPs: *N* = 19	3 SPs: *N* = 8	3 SPs: *N* = 11

All variables other than the number of SPs were included as covariates in regression models. Data on school size, geography, and free/reduced lunch were derived from baseline school administrator surveys and/or state data sources (e.g., MI School Data; https://www.mischooldata.org/); SP tenure and education from SP baseline surveys; and pre-randomization CBT delivery from SP weekly CBT reports during the pre-randomization run-in phase

**Table 4:** School-level characteristics for phase 2 randomization (Facilitation vs. no Facilitation) for slower-responder schools (*N* = 83 schools)

	All slower-responder schools (***N*** = 83)	No Facilitation (***N*** = 42)	Facilitation (***N*** = 41)
**Proportion of schools with >500 students (vs. ≤ 500 students)**	0.65	0.67	0.63
**Proportion of rural schools (vs. non-rural)**	0.55	0.55	0.56
**Proportion of schools with >50% students on free/reduced lunch program (vs. ≤ 50%)**	0.37	0.40	0.34
**Proportion of schools with any pre-randomization CBT delivery (vs. none)**	0.53	0.50	0.56
**Proportion of schools with top 50% total CBT sessions in the 8 weeks within Phase 1 arm**	0.49	0.48	0.51
**Coaching (vs. no Coaching)**	0.49	0.50	0.49
**School-level proportion of SPs with graduate degree: mean (SD)**	0.88 (.27)	0.81 (.35)	0.96 (.14)
**Number of SPs per school**	Mean = 1.86	Mean = 1.90	Mean = 1.80
	1 SP: *N* = 31	1 SP: *N* = 16	1 SP: *N* = 15
	2 SPs: *N* = 33	2 SPs: *N* = 14	2 SPs: *N* = 19
	3 SPs: *N* = 19	3 SPs: *N* = 12	3 SPs: *N* = 7

All variables other than the number of SPs were included as covariates in regression models. Data on school size, geography, and free/reduced lunch were derived from baseline school administrator surveys and/or state data sources (e.g., MI School Data; https://www.mischooldata.org/); SP tenure and education from SP baseline surveys; and pre-randomization CBT delivery from SP weekly CBT reports during the pre-randomization run-in phase

At least one SP from all 94 schools remained in the study through completion; however, one SP dropped out of the study during phase 2b (Fig. 5). SPs completed 4720 of 7267 possible weekly CBT reports (65%) and a median of 32 weeks (interquartile range: 18–40).

### Primary outcome: average total CBT delivery

CBT delivery increased across all groups: 154 of 169 SPs (91%) reported delivering CBT at least once during the 43-week post-randomization period, and 20,517 CBT sessions were reported by SPs during this period. Estimated average total CBT delivery by strategy ranged from 94.53 (REP + Coaching) to 154.06 (REP + Facilitation) sessions (Table 5).

**Table 5:** Average CBT delivery (primary outcome), by phase and total

Implementation strategy	By Study Phase				Average total CBT delivered (43 weeks)
	Phase 1 (9 weeks)	Phase 2a (11 weeks)	Phase 2b (13 weeks)	Phase 3 (10 weeks)	
**REP**	22.28 (16.90, 27.67)	31.70 (20.77, 42.62)	38.17 (26.17, 50.17)	28.93 (18.03, 39.83)	121.08 (87.52, 154.65)
**REP + Facilitation**		38.07 (28.43, 47.71)	49.86 (35.55, 64.17)	43.85 (29.03, 58.66)	154.06 (109.14, 198.97)
**REP + Coaching**	15.32 (11.80, 18.84)	26.05 (20.28, 31.82)	29.92 (22.15, 37.69)	23.23 (14.18, 32.29)	94.53 (76.53, 112.53)
**REP + Coaching+Facilitation**		28.06 (21.23, 34.88)	37.44 (27.49, 47.40)	30.58 (19.88, 41.28)	111.40 (86.63, 136.16)

95% confidence intervals in parentheses. For phase 1, only two estimates are shown as all groups were consistent with either REP alone (first and second rows) or REP + Coaching (third and fourth rows)

Pairwise comparisons for average total CBT delivery are shown in Table 6. For ASIC’s primary aim, there is insufficient evidence to reject the null hypothesis that there is no difference in average total CBT delivery between REP alone and the REP + Coaching + Facilitation adaptive implementation strategy (estimate = 9.69; 95% CI: −30.03, 49.40; *p*-value = 0.63). Consistent with this finding, the estimated effect is very small (effect size = 0.08).

**Table 6:**
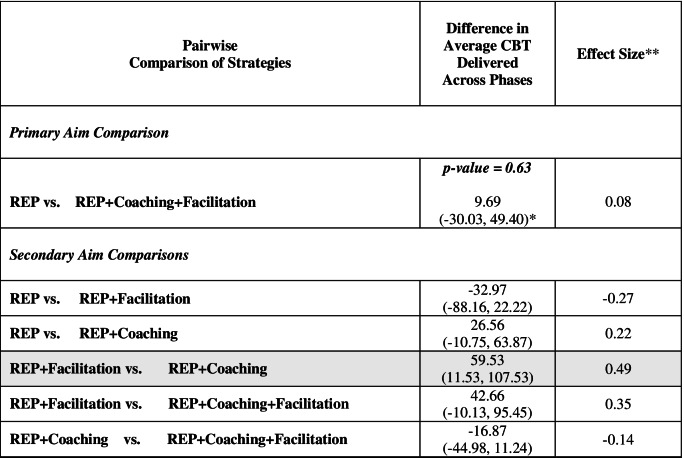
Pairwise comparisons for total CBT delivery (primary outcome)

*95% confidence intervals in parentheses

**Effect size was calculated based on a standard deviation of 120.8 for Total CBT Delivery. The shaded row indicates the largest effect size

Estimated effects were larger for ASIC’s secondary aim comparisons. SPs at schools assigned to REP + Facilitation delivered an estimated average of 59.53 more CBT sessions than SPs at schools assigned to REP + Coaching (95% CI: 11.53, 107.53; effect size = 0.49) and 42.66 more sessions than REP + Coaching + Facilitation (95% CI: −10.13, 95.45; effect size = 0.35). SPs at schools assigned to REP delivered an estimated 32.97 fewer sessions than SPs at schools assigned to REP + Facilitation (95% CI: −88.16, 22.22; effect size = −0.27) but 26.56 more sessions than SPs at schools assigned REP + Coaching (95% CI: −10.75, 63.87; effect size = 0.22). Using an effect size equal to or below the absolute value of 0.1 to suggest no clinically significant difference between strategies, Table 7 shows the hypothesized versus estimated order for the implementation strategies.

**Table 7:** Hypothesized vs. estimated order for implementation strategies

***Hypothesized order***	REP + Coaching + Facilitation > REP + Coaching = REP + Facilitation > REP
***Estimated order***	REP + Facilitation > REP = REP + Coaching + Facilitation > REP + Coaching

### Secondary outcomes: average total CBT delivery by type

Estimated average total CBT delivery by type ranged from 16.81 group sessions (REP alone) to 81.60 individual brief sessions (REP + Facilitation) (Table 8). Across all strategies, the greatest percentage of average total CBT delivery were individual brief sessions (≥43%) and the lowest group sessions (≤19%). Pairwise comparisons for average total CBT delivery by type (Table 9) and estimated ordering of strategies by type (Table 10) are also shown.

**Table 8:**
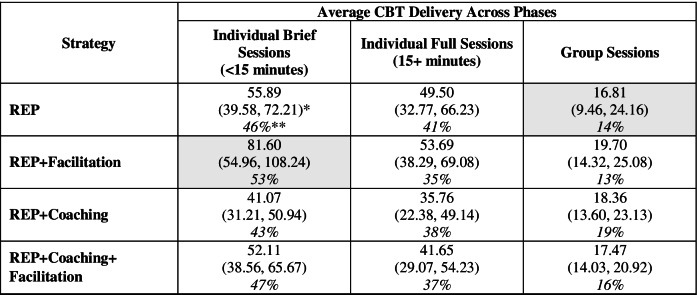
Average CBT delivery, by type (secondary outcomes)

*95% confidence intervals in parentheses

**Indicates percent of total CBT delivery within strategy. The highest and lowest percentage cells across all strategies are shaded

**Table 9:**
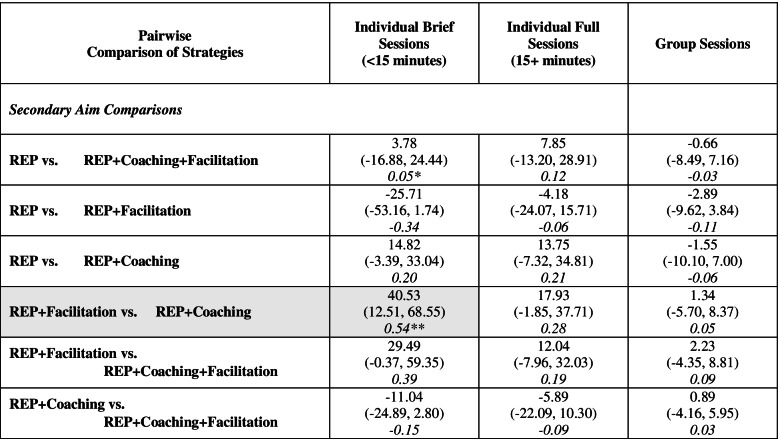
Pairwise comparisons for average CBT delivery, by type (secondary outcomes)

*Effect sizes were calculated based on the following estimated standard deviations: 75.5 for individual brief Sessions, 64.8 for individual full sessions, 25.5 for group sessions. **Shaded cell indicates the largest effect size

**Table 10:** Estimated ordering for implementation strategies for CBT delivery, by type

***Individual brief sessions***	REP + Facilitation > REP = REP + Coaching + Facilitation > REP + Coaching
***Individual full sessions***	REP + Facilitation = REP > REP + Coaching + Facilitation = REP + Coaching
***Group sessions***	REP + Facilitation > REP = REP + Coaching + Facilitation = REP + Coaching

## Discussion

This study compared four different implementation strategies on the total number of SP-reported CBT sessions delivered. The REP and REP + Coaching strategies were non-adaptive, offering the same types of support to all schools across all phases; the REP + Facilitation and REP + Coaching + Facilitation strategies were adaptive, augmenting with Facilitation in phase 2 for slower-responder schools. With respect to our primary aim hypothesis, we found no evidence of differences in average total CBT delivery between REP and REP + Coaching + Facilitation. In secondary aim comparisons, we found that across the four strategies, the adaptive REP + Facilitation strategy resulted in the highest average CBT delivery (154.1 sessions per SP) and the non-adaptive REP + Coaching strategy the lowest (94.5 sessions per SP). Examining CBT delivery by type, however, most differences across strategies reflected higher vs. lower reports of brief (≤15 min) individual CBT sessions.

### Harnessing schools and school professionals for improving adolescent mental health

This manuscript adds to the growing literature supporting the potential for SPs to help fill gaps in adolescent access to mental health care by offering mental health EBPs, like CBT, in schools. Indeed, all strategies, including the low-intensity REP alone strategy, showed high levels of CBT uptake, with more than 90% of participating SPs (i.e., that completed training) reporting CBT delivery at least once (relative to CBT adoption rates of 35–40% elsewhere [90]). Combined, SPs delivered more than 20,000 CBT sessions over 43 weeks. Furthermore, as our secondary trend analyses show (Supplementary Appendix C), under all strategies CBT delivery remained consistent or increased across all study phases, including the maintenance phase, which followed discontinuation of Coaching and/or Facilitation.

This study also provides support for the feasibility of organizations like TRAILS offering adaptive implementation strategies in schools. As noted, TRAILS was exclusively responsible for monitoring response status after phase 1 and, as applicable, adapting implementation support in phase 2.

### Explaining differences across implementation strategies

Although prior research has drawn attention to potential shortcomings of offering intensive implementation support, especially in lower-resourced settings [40, 91], our team was nonetheless surprised to not find support for our hypothesis that, for our primary outcome of average total CBT delivery, the most intensive strategy (REP + Coaching + Facilitation) would outperform the least intensive strategy (REP). Also surprising was that the REP + Facilitation strategy, which adapted phase 2 support based on response to phase 1, differed markedly from the non-adaptive REP + Coaching strategy for three of the four outcomes. Note, however, that these analyses focus on SP-reported CBT delivery; future analyses will examine other outcomes, including CBT fidelity and change in student mental health outcomes. However, these initial findings raise several potential explanations.

While future analyses will examine mechanisms more systematically, we postulate that much of the benefit of REP + Facilitation is due to the fact that Facilitation was provided only to schools identified as slower responders, i.e., in context of a recognized need. The salience of the targeted barriers and/or the perceived appropriateness of Coaching and Facilitation strategies may also have differed. SPs may have had concerns about Coach attendance at student CBT sessions risking student privacy or confidence or may have felt they did not need further CBT skill development. Facilitation, which has proven effective in other implementation trials [36, 40, 92, 93], also provides support that is highly tailored to specific school needs [80, 94, 95] by “bundling” other discrete implementation strategies [80, 94, 96, 97], and generally addresses barriers that were more external and jointly identified with the SP, thus not risking SP concerns regarding student privacy or perceptions of help-seeking. Prior TRAILS’ Coaching studies have not reported such concerns [26]; however, these studies largely recruited SPs motivated to receive Coaching. In contrast, ASIC recruited a more heterogeneous sample of SPs interested in receiving support for implementing CBT, but not necessarily via Coaching.

Lower SP engagement also suggests that Coaching may have led to greater real or perceived burden by SPs, relative to Facilitation. Coaching is typically offered in the context of CBT groups, which requires SPs to identify and coordinate student CBT groups and align group delivery with Coaching visits. As both SPs and Coaches were balancing many time commitments, this coordination may have lessened SP engagement with Coaching. Facilitation also includes some scheduling burden, but is done primarily over the phone and was also not dependent on student CBT group coordination. Furthermore, Facilitation’s scheduling burden may have been more acceptable to SPs given their awareness that Facilitation was offered based on a recognized need for further support.

Facilitation was also provided by a single Facilitator, ensuring strategy consistency and fidelity, while Coaching was provided by 42 existing providers across Michigan who were employed by other local agencies near the schools they served. While Coaches were required to complete Coach training through TRAILS [66], there was likely variability in the quality of Coaching provided, as well as in Coach commitment to their schools given other responsibilities.

### Limitations

First, our results rely on SP self-reported CBT delivery. Self-report of implementation outcomes like adoption, reach, and even fidelity is common in implementation studies [98], especially in lower-resourced settings [40, 41], including schools [99, 100]. As clinicians (e.g., social workers, counselors, psychologists), SPs were also accustomed to documenting mental health services, and the process for documenting CBT delivery was identical across arms. However, it is possible that SPs assigned to Coaching could have reported a lower number of CBT sessions if Coach feedback led to different perceptions of what counted as CBT, given Coaching’s explicit focus on improving CBT knowledge and expertise. However, we took proactive steps to protect against this, including (i) clearly explaining to all SPs prior to randomization how we were defining (and how they should be reporting) CBT delivery for research purposes and (ii) having SPs “practice” reporting CBT during the pre-randomization REP-only phase to ensure their comfort and consistency prior to offering any additional support (e.g., Coaching, Facilitation). SPs’ reporting CBT components delivered each week (Fig. 3) also likely protected against SPs reporting *any* mental health-focused interaction as CBT. Finally, as reported above, weekly CBT response rates did not vary across Coaching vs. no Coaching study arms.

Second, generalizability is limited to schools in Michigan that had a TRAILS Coach in their vicinity, but nonetheless included a diverse group of urban, suburban, and rural communities.

### Future work

Future manuscripts will examine differences across strategies for two key secondary outcomes: CBT fidelity/component delivery and student mental health. We will also examine moderators of effectiveness for Coaching and (among slower-responder schools) Facilitation, including different definitions of “slower-responders” for purposes of deciding how to best tailor Facilitation (vs. no Facilitation). These analyses will help to inform a more fully tailored adaptive implementation strategy for efficiently scaling up SP-delivered CBT in schools by matching implementation strategies to identified barriers [101] or short-term implementation outcomes [41]. Qualitative interviews and strategy (e.g., Facilitation) tracking data will also be used to investigate mechanisms of effectiveness (e.g., strategy burden, need, adaptability), whether strategies addressed intended barriers (e.g., CBT knowledge, organizational barriers), and sustainability of SP-delivered CBT during and after COVID-19.

## Conclusion

As the COVID-19 pandemic continues to shed light on the role schools and SPs play in student mental health, questions abound as to which implementation strategies are most effective at addressing barriers to offering EBPs like CBT at scale. Our findings suggest that, among the four strategies examined, the most effective strategy for increasing average SP CBT delivery is a two-phase adaptive implementation strategy that (i) offers REP (a low-intensity, low-cost strategy) in phase 1 to all schools and, in phase 2, (ii) augments REP with Facilitation for slower-responder schools and (iii) continues REP for schools that respond to REP.

## Supplementary Information

**Additional file 1: Appendix A.** School Professional Assessment Survey. **Appendix B.** School Professional Characteristics and Background. **Appendix C.** Re-Analysis Focusing on CBT Delivery Trends. **Appendix D.** Missing Data and Imputation.

## Data Availability

The datasets used and/or analyzed during the current study are available from the corresponding author on reasonable request.

## Abbreviations

ASIC: Adaptive School-based Implementation of CBT
CBT: Cognitive behavioral therapy
CMH: Community mental health
EBP: Evidence-based practice
i-PARIHS: Integrated-Promoting Action on Research Implementation in Health Services
PBIS: Positive Behavior Interventions and Supports
QUERI: Quality Enhancement Research Initiative
REP: Replicating Effective Programs
SMART: Sequential, Multiple-Assignment Randomized Trial
SP: School professional
TRAILS: Transforming Research into Action to Improve the Lives of Students
