# Correction: Primary aim results of a clustered SMART for developing a school-level, adaptive implementation strategy to support CBT delivery at high schools in Michigan

**DOI:** 10.1186/s13012-022-01229-0

**Published:** 2022-08-11

**Authors:** Shawna N. Smith, Daniel Almirall, Seo Youn Choi, Elizabeth Koschmann, Amy Rusch, Emily Bilek, Annalise Lane, James L. Abelson, Daniel Eisenberg, Joseph A. Himle, Kate D. Fitzgerald, Celeste Liebrecht, Amy M. Kilbourne

**Affiliations:** 1 Department of Health Management and Policy, School of Public Health, University of Michigan, SPH II, 1415 Washington Heights, Ann Arbor, MI 48109 USA; 2 Department of Psychiatry, Michigan Medicine, University of Michigan, Ann Arbor, USA; 3 Survey Research Center, Institute of Social Research, University of Michigan, Ann Arbor, USA; 4 Department of Statistics, University of Michigan, Ann Arbor, USA; 5 Department of Health Policy and Management, UCLA, Los Angeles, USA; 6 School of Social Work, University of Michigan, Ann Arbor, USA; 7 Department of Psychiatry, Columbia University Irving Medical Center/New York State Psychiatric Institute, New York City, USA; 8 Department of Learning Health Sciences, Michigan Medicine, University of Michigan, Ann Arbor, USA; 9 Quality Enhancement Research Initiative (QUERI), US Department of Veterans Affairs, Washington, DC USA

**Correction: Implement Sci 17, 42 (2022)**

**https://doi.org/10.1186/s13012-022-01211-w**

Following publication of the original article [1], it was reported that an incorrect version of Additional file 1 was published. The correct files are included in this Correction and the original article [1] has been updated.

## Supplementary Information

**Additional file 1: Appendix A**. School Professional Assessment Survey. **Appendix B**. School Professional Characteristics and Background. **Appendix C**. Re-Analysis Focusing on CBT Delivery Trends. **Appendix D**. Missing Data and Imputation.
