# Supplementary material for: Primary aim results of a clustered SMART for developing a school-level, adaptive implementation strategy to support CBT delivery at high schools in Michigan
Source: Implement Sci. 2022 Jul 8;17:42. doi: 10.1186/s13012-022-01211-w (PMC9264291; doi:10.1186/s13012-022-01211-w)
Supplement: Supplementary file 1 — Additional file 1: Appendix A. School Professional Assessment Survey. Appendix B. School Professional Characteristics and Background. Appendix C. Re-Analysis Focusing on CBT Delivery Trends. Appendix D. Missing Data and Imputation. [file 13012_2022_1211_MOESM1_ESM.zip › Appendix A. School Professional Assessment Survey_ESM.pdf]

## Appendix A. School Professional Assessment Survey

1. Since attending TRAILS CBT training, please select the CBT components you have delivered to any students (select all that apply)

a. Psychoeducation
b. Relaxation
c. Exposure
d. Behavioral Activation
e. Cognitive Restructuring/ Coping
f. None

2. (If 3 or more components were chosen) To how many students (if any) have you delivered 3 or more CBTcomponents? __________

3. In your school, do you believe any of the following are barriers to your delivering CBT to your students? Please select all that apply. If none of these are applicable, please select "None of these are applicable."

| | **Select if applicable** |
|---|---|
| Previous delivery of CBT has not been associated with improved student outcomes | |
| Low confidence in identifying students that might benefit from CBT | |
| Low acceptance among other school professionals or colleagues | |
| Lack of support from school administrators | |
| Lack of students that might benefit from CBT | |
| Low confidence in my ability to deliver CBT effectively | |
| Too many other responsibilities or demands on my time | |
| Lack of physical resources (e.g., a room for group CBT delivery) | |
| I don't believe CBT is effective for improving student mental health | |
| My efforts to engage students in CBT have been ineffective | |
| **None of these are applicable** | |
