# Supplementary material for: Primary aim results of a clustered SMART for developing a school-level, adaptive implementation strategy to support CBT delivery at high schools in Michigan
Source: Implement Sci. 2022 Jul 8;17:42. doi: 10.1186/s13012-022-01211-w (PMC9264291; doi:10.1186/s13012-022-01211-w)
Supplement: Supplementary file 1 — Additional file 1: Appendix A. School Professional Assessment Survey. Appendix B. School Professional Characteristics and Background. Appendix C. Re-Analysis Focusing on CBT Delivery Trends. Appendix D. Missing Data and Imputation. [file 13012_2022_1211_MOESM1_ESM.zip › Appendix B. School Professional Characteristics and Background_ESM.pdf]

Appendix B. School Professional Characteristics & Background

Table B.1 School Professional (SP) characteristics (n=169)

**Characteristics**	**% (n)/Mean (SD; Range)**
**Years in current position**	8.0 years (SD = 7.7; 0-45)
**Professional Role**	
School Counselor	59.2% (n=100)
School Social Worker	23.1% (n=39)
Other[a]	17.8% (n=30)
**Race**	
American Indian or Alaska Native	1.8% (n=3)
Asian	0.6% (n=1)
Bi-racial or multi-racial	1.8% (n=3)
Black or African American	1.2% (n=2)
White or Caucasian	89.3% (n=151)
Did not disclose	5.3% (n=9)
**Gender**	
Female	84.6% (n=143)
Male	11.8% (n=20)
Did not disclose	3.6% (n=6)
**Highest Educational Attainment**	
Bachelor or Associate degree	6.5% (n=11)
Master of Social Work	24.9% (n=42)
Master (or Specialist) in Counseling/School Counseling	57.4% (n=97)
Master (or Specialist) in Psychology/School Psychology	3.0% (n=5)
Master (or Specialist) in Education	3.0% (n=5)
Other Master (or Specialist)	1.8% (n=3)
Doctoral degree	0.6% (n=1)
Other degrees/did not disclose	3.0% (n=5)
**Caseload size**	296 students (SD=249; 15-1850)
**Caseload population**	
Not specific to any population	40.2% (n=68)
Exclusively or primarily students in general education	34.9% (n=59)
Exclusively or primarily students in special education	20.7% (n=35)
Did not disclose	4.1% (n=7)
**Prior CBT training**	
Yes, formal (i.e., graduate program)	33.7% (n=57)
Yes, informal only	26.0% (n=44)
No	36.7% (n=62)
Did not disclose	3.6% (n=6)

SD = Standard deviation

[a]: Other reported roles include school psychologist (n=5), behavioral intervention specialist (n=4), special education teacher (n=4), general education teacher (n=4), school success worker (n=4), administrator (n=3), school nurse (n=1), wellness therapist/coordinator (n=1) and other (n=4).

Table B.2 SP prior training on CBT in graduate school (i.e., formal training) (n=169)

**CBT training in graduate school**	
No graduate training	62.7% (n=106)
1-2 lectures on CBT as part of a larger course	30.8% (n=52)
Quarter/semester course exclusively on CBT	2.4% (n=4)
Didactic course on CBT with associated clinical supervision of one or more cases	0.6% (n=1)
Did not disclose	3.6% (n=6)

Table B.3 SP prior training on CBT in the past 5 years (i.e., informal training) (n=169)

**Professional development on CBT in the past 5 years (Choose all that apply)**	
None	50.3% (n=85)
Brief presentation or workshop	26.0% (n=44)
Self-directed (e.g., online course, reading, YouTube videos)	24.3% (n=41)
1-to-2-day training	9.5% (n=16)
Mentioned in other classes/trainings	4.1% (n=7)
Training with follow-up consultation or supervision of a case	3.6% (n=6)
Did not disclose	4.1% (n=7)
