# Supplementary material for: Primary aim results of a clustered SMART for developing a school-level, adaptive implementation strategy to support CBT delivery at high schools in Michigan
Source: Implement Sci. 2022 Jul 8;17:42. doi: 10.1186/s13012-022-01211-w (PMC9264291; doi:10.1186/s13012-022-01211-w)
Supplement: Supplementary file 1 — Additional file 1: Appendix A. School Professional Assessment Survey. Appendix B. School Professional Characteristics and Background. Appendix C. Re-Analysis Focusing on CBT Delivery Trends. Appendix D. Missing Data and Imputation. [file 13012_2022_1211_MOESM1_ESM.zip › Appendix C. Re-Analysis Focusing on CBT Delivery Trends_ESM.pdf]

Appendix C. Re-Analysis Focusing on CBT Delivery Trends

Phases 1, 2a, 2b and 3 of the study varied slightly in length, ranging from 9 to 13 weeks. This feature of the study does not present challenges to comparing average total CBT delivery between two or more embedded strategies (e.g., as in the study's primary aim). However, there may be secondary interest in comparing average total CBT delivery *across* phases of the study (as well as *between* embedded strategies), i.e., to understand different trends over time across embedded strategies. To answer this new scientific question concerning trends in CBT delivery, it may be more desirable to focus on a measure of "average CBT delivery per week". In this Appendix C, we report the results of a re-analysis of the data with this goal in mind. Specifically, for each embedded strategy at each phase, we computed the following ratio:

- the average total CBT delivered at each phase (as defined and reported in the manuscript's main narrative), divided by
- the total number of weeks in the phase.

Results of a Re-analysis Focusing on Trends Across Phases

Results are reported in Supplementary Appendix Table C1 and Supplementary Appendix Figure C1. All strategies led to increases in estimated CBT delivery per week from Phase 1 to 2a; REP+Facilitation schools continued to see small increases in delivery into Phases 2b and 3 while delivery under other strategies (REP, REP+Coaching, REP+Coaching+Facilitation) remained largely flat.

Supplementary Appendix Table C1.
Weekly Average Total CBT Delivery (Primary Outcome), by Phase

Implementation Strategy	By Study Phase: Phase 1 (9 weeks)	Phase 2a (11 weeks)	Phase 2b (13 weeks)	Phase 3 (10 weeks)	Average CBT Delivered Across Phases (43 weeks)
**REP**	2.48 (1.88, 3.07)	2.88 (1.89, 3.87)	2.94 (2.01, 3.86)	2.89 (1.80, 3.98)	2.82 (2.04, 3.60)
**REP+Facilitation**		3.46 (2.58, 4.34)	3.84 (2.73, 4.94)	4.38 (2.90, 5.87)	3.58 (2.54, 4.63)
**REP+Coaching**	1.70 (1.31, 2.09)	2.37 (1.84, 2.89)	2.30 (1.70, 2.90)	2.32 (1.42, 3.23)	2.20 (1.78, 2.62)
**REP+Coaching+ Facilitation**		2.55 (1.93, 3.17)	2.88 (2.11, 3.65)	3.06 (1.99, 4.13)	2.59 (2.01, 3.17)

Note: 95% confidence intervals in parentheses. For Phase 1, only two estimates are shown as all groups were consistent with either REP only (first and second rows) or REP + Coaching (third and fourth rows). The numbers presented here are the estimates shown in Table 3 of the main manuscript, divided by the number of weeks in each phase.

Figure C1. Weekly Average Total CBT Delivery (Primary Outcome), by Phase

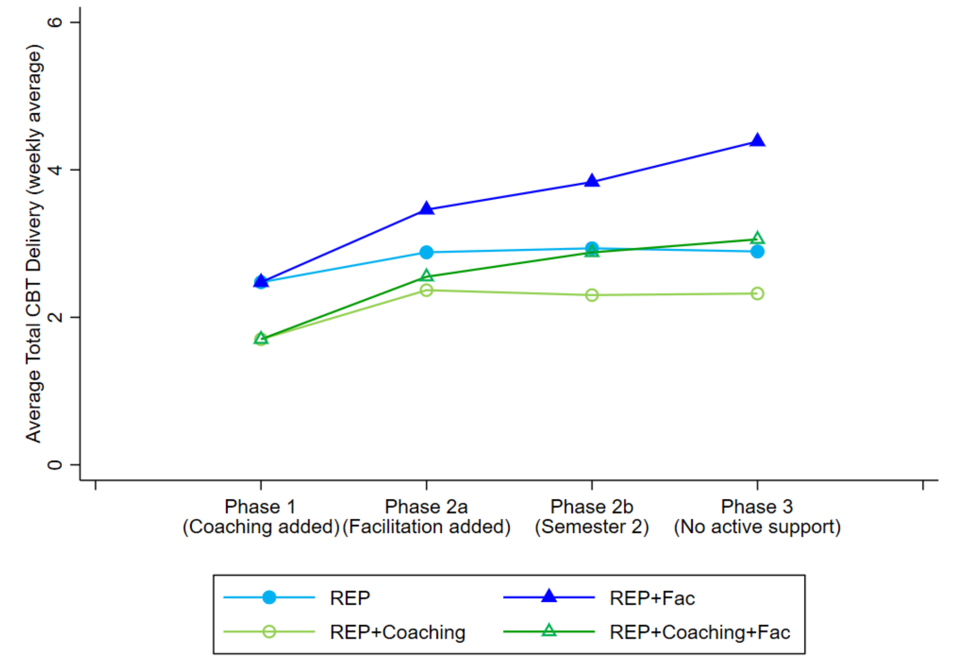

Note: Each line corresponds to one of four embedded strategies (Table C1). The y-axis is an estimate of the average number of CBT sessions delivered per week. REP = Replicating Effective Programs; Fac = Facilitation.

Secondary outcomes: Trends in CBT delivery by type

Results for trends in our secondary outcomes of CBT delivery by type (i.e., estimated total number of Groups, Full Individual and Brief Individual CBT sessions delivered by SPs under each embedded implementation strategy) using by-phase weekly averages are shown in Supplementary Appendix Table C2 and Supplementary Appendix Figure C2. Trends for delivery of Brief Individual CBT sessions (Table C2.A; Figure C2, Panel A) are similar to trends for Weekly Average Total CBT, described above. Trends for Full Individual sessions (Table C2.B; Figure C2, Panel B) did not vary markedly across strategies, and show the largest increases between Phases 2a and 2b, with all strategies largely maintaining weekly delivery in Phase 3. Group delivery by week (Table C2.C, Figure C2, Panel C) increased between Phase 1 and Phase 2a, and declined slightly through Phases 2b and 3.

Table C2. Weekly Average CBT delivery by Type (Secondary Outcomes), by Phase

Table C2.A. Weekly Average Brief Individual CBT delivery, by Phase

Implementation Strategy	By Study Phase				Average CBT Delivered Across Phases (43 weeks)
	Phase 1 (9 weeks)	Phase 2a (11 weeks)	Phase 2b (13 weeks)	Phase 3 (10 weeks)	
**REP**	1.14 (0.73, 1.55)	1.22 (0.70, 1.74)	1.40 (0.88, 1.92)	1.40 (0.81, 1.99)	1.30 (0.92, 1.68)
**REP+Facilitation**		1.83 (1.11, 2.55)	2.00 (1.21, 2.78)	2.52 (1.33, 3.71)	1.90 (1.28, 2.52)
**REP+Coaching**	0.66 (0.43, 0.89)	1.06 (0.71, 1.40)	0.91 (0.61, 1.21)	1.17 (0.78, 1.55)	0.96 (0.73, 1.18)
**REP+Coaching+ Facilitation**		1.15 (0.77, 1.53)	1.29 (0.84, 1.74)	1.67 (1.05, 2.29)	1.21 (0.90, 1.53)

Table C2.B. Weekly Average Full Individual CBT delivery, by Phase

Implementation Strategy	By Study Phase				Average CBT Delivered Across Phases (43 weeks)
	Phase 1 (9 weeks)	Phase 2a (11 weeks)	Phase 2b (13 weeks)	Phase 3 (10 weeks)	
**REP**	0.94 (0.66, 1.22)	1.14 (0.64, 1.64)	1.28 (0.83, 1.74)	1.18 (0.66, 1.70)	1.15 (0.76, 1.54)
**REP+Facilitation**		1.07 (0.71, 1.44)	1.44 (0.95, 1.94)	1.46 (0.85, 2.07)	1.25 (0.89, 1.61)
**REP+Coaching**	0.66 (0.36, 0.95)	0.66 (0.32, 1.00)	0.97 (0.54, 1.40)	0.99 (0.17, 1.82)	0.83 (0.52, 1.14)

**REP+Coaching+ Facilitation**		0.88<br>(0.55, 1.21).	1.19<br>(0.73, 1.65)	1.05<br>(0.46, 1.65)	0.97<br>(0.68, 1.26)

Table C2.C. Weekly Average Group CBT delivery, by Phase

**Implementation Strategy**	**By Study Phase**				**Average CBT Delivered Across Phases (43 weeks)**
	**Phase 1 (9 weeks)**	**Phase 2a (11 weeks)**	**Phase 2b (13 weeks)**	**Phase 3 (10 weeks)**	
**REP**	0.39<br>(0.19, 0.60)	0.58<br>(0.32, 0.85)	0.28<br>(0.07, 0.50)	0.32<br>(0.01, 0.62)	0.39<br>(0.22, 0.56)
**REP+Facilitation**		0.57<br>(0.41, 0.73)	0.45<br>(0.26, 0.63)	0.41<br>(0.24, 0.59)	0.46<br>(0.33, 0.58)
**REP+Coaching**	0.38<br>(0.25, 0.51)	0.69<br>(0.44, 0.94)	0.41<br>(0.22, 0.60)	0.20<br>(0.11, 0.29)	0.43<br>(0.32, 0.54)
**REP+Coaching+ Facilitation**		0.51<br>(0.38, 0.64)	0.40<br>(0.28, 0.51)	0.32<br>(0.19, 0.46)	0.41<br>(0.33, 0.49)

Figure C2. CBT Delivery, by Type (Secondary Outcomes) and Phase

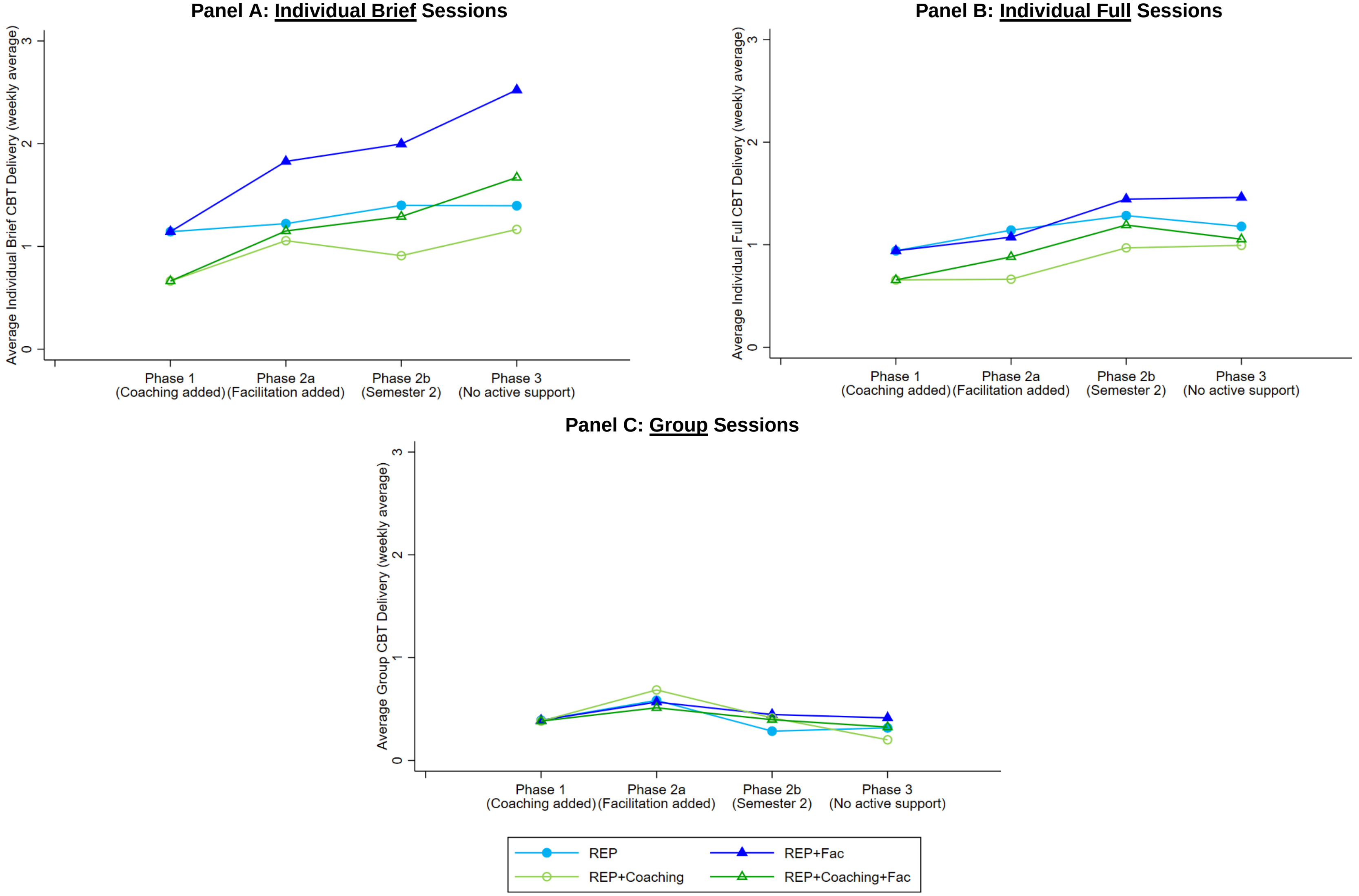

Estimated average weekly delivery computed as estimated average delivery for each phase divided by the number of weeks in the phase. Brief individual sessions were defined as less than 15 minutes; full individual sessions as greater than 15 minutes. REP = Replicating Effective Programs; Fac = Facilitation.
