# Supplementary material for: Primary aim results of a clustered SMART for developing a school-level, adaptive implementation strategy to support CBT delivery at high schools in Michigan
Source: Implement Sci. 2022 Jul 8;17:42. doi: 10.1186/s13012-022-01211-w (PMC9264291; doi:10.1186/s13012-022-01211-w)
Supplement: Supplementary file 1 — Additional file 1: Appendix A. School Professional Assessment Survey. Appendix B. School Professional Characteristics and Background. Appendix C. Re-Analysis Focusing on CBT Delivery Trends. Appendix D. Missing Data and Imputation. [file 13012_2022_1211_MOESM1_ESM.zip › Appendix D. Missing Data and Imputation _ESM.pdf]

Appendix D. Missing Data and Imputation

As explained in the main narrative, multiple imputation was used to replace missing values in the outcomes and other measures [1]. Forty data sets were generated. All estimates, standard errors, and hypothesis tests reported below were calculated using standard rules [2], [3] for combining the results of identical analyses performed on each of the 40 imputed data sets. This Supplementary Appendix describes additional details related to the missing data and imputation approach used. We also report the study results without the multiply imputed data. This was done to document/understand whether there were any discrepancies in the results when analyzed with and without multiple imputation.

Missing Data Summary

Recall that a total of 169 SPs at 94 high schools entered the study. All 169 participating SPs had complete data for all six baseline, school-level covariates: school size, location of school, percentage of students on free/reduced lunch program, and any pre-randomization delivery of CBT, as well as school-aggregated SP education and job tenure, so no imputation was necessary for these variables. In terms of weekly CBT delivery reports (from which the primary outcome was constructed), overall SPs completed 4,720 of 7,267 possible weekly CBT reports during the 43-week study, for an overall response rate of 65% (i.e., 65% of 169 reports/week x 43 weeks). On average, SPs completed a median of 32 of 43 weekly CBT reports (interquartile range: 18 to 40).

Imputation of Structural Zeros

Although weekly CBT reports were paused during known school holidays (e.g., winter and summer holidays), some weekly reports were requested when no CBT delivery was possible for a particular SP—for example, when a school was on a mid-winter or spring break or when an SP was on maternity or medical leave (and informed the study team thereof). SPs in these situations were still encouraged (and incentivized) to report no CBT delivery for these weeks, but in some cases they did not, and the data appeared as missing. In these known cases, weekly CBT delivery was imputed as 0. These known structural zeroes accounted for 53 SP weekly CBT delivery reports (or 0.7% of 7,267 possible CBT delivery reports).

Multiple Imputation

The remaining missing weekly CBT delivery reports (2,494 of 7,267, or 34.3%) were multiply imputed using Stata Version 16 [4] with the multiple imputation by chained equations (MICE) program [5], [6]. For each missing weekly CBT report, values for three CBT delivery measures were imputed: (i) the number of individual brief sessions delivered, (ii) the number of individual full sessions delivered, and (iii) the number of group sessions delivered. A different imputation model was specified for each CBT delivery measure for each week of Phases 1, 2a, 2b and 3. The week *t* model included the following predictors: two baseline, school-level variables (school size and percentage of students on free/reduced lunch program), a summary of the total CBT delivered in the prior phase (i.e., the sum of the CBT delivered across all weeks in the prior phase), and past or current implementation strategies to which the school was assigned (e.g., Coaching, Facilitation). Total CBT delivered in week *t* was then computed as the sum of the three imputed variables.

Analyses Using the Incomplete Data

For comparison, the results using the incomplete (i.e., not imputed) data are presented in Tables D1-D4. Unlike the more appropriate analysis using the multiply imputed data reported in the manuscript's main narrative, this "complete case analysis" removes (i.e., drops) missing SP-weeks from the analysis. Findings show, as expected: (i) estimates of the average total number of CBT sessions reported are lower using incomplete data than with the multiply imputed data; and (ii) confidence intervals are slightly larger using the incomplete data than with the multiply imputed data. However, the signs of all effect estimates are in the same direction and study inferences generally do not differ from the results and conclusions reported in the main narrative using the multiply imputed data.

Table D1. Total CBT Delivery (Primary Outcome), by Phase and Across Phases, using Incomplete Data

Implementation Strategy	By Study Phase				Average CBT Delivered Across Phases (43 weeks)
	Phase 1 (9 weeks)	Phase 2a (11 weeks)	Phase 2b (13 weeks)	Phase 3 (10 weeks)	
REP	18.60 (13.59, 23.61)	24.74 (13.73, 35.74)	25.78 (12.33, 39.23)	21.28 (8.78, 33.77)	90.39 (55.78, 125.01)
REP+Facilitation		26.97 (17.38, 36.56)	36.51 (21.66, 51.36)	33.68 (19.17, 48.19)	115.75 (78.52, 152.98)
REP+Coaching	11.61 (8.32, 14.89)	16.80 (11.71, 21.89)	22.14 (14.51, 29.78)	15.32 (8.78, 21.85)	65.86 (47.87, 83.86)
REP+Coaching+ Facilitation		18.51 (11.49, 25.53)	24.48 (13.39, 35.58)	20.09 (7.68, 32.50)	74.69 (48.96, 100.42)

*95% confidence intervals in parentheses. See Table 4 in main narrative for comparison

Table D2. Pairwise Comparisons for Total CBT Delivery (Primary Outcome), using Incomplete Data

Pairwise Comparison of Strategies	Difference in Average CBT Delivered Across Phases	Effect Size**
Primary Aim Comparison		
**REP vs. REP+Coaching +Facilitation**	***p-value = 0.46***<br>15.70<br>(-25.87, 52.27)*	0.13
Secondary Aim Comparisons		
**REP vs. REP+Facilitation**	-25.36<br>(-73.06, 22.34)	-0.21
**REP vs. REP+Coaching**	24.53<br>(-13.74, 62.80)	0.20
**REP+Facilitation vs. REP+Coaching**	49.89<br>(11.43, 88.34)	0.41
**REP+Facilitation vs. REP+Coaching +Facilitation**	41.06<br>(-3.23, 85.35)	0.34
**REP+Coaching vs. REP+Coaching +Facilitation**	-8.83<br>(-36.06, 18.40)	-0.07

*95% confidence intervals in parentheses. **Effect size was calculated based on a standard deviation of 121.2 for Total CBT Delivery. The cell with the largest effect size is shaded. See Table 5 in main narrative for comparison

Table D3. CBT Delivery, by Type (Secondary Outcomes), using Incomplete Data

**Strategy**	**Average CBT Delivery Across Phases**		
	**Individual Brief Sessions (<15 minutes)**	**Individual Full Sessions (15+ minutes)**	**Group Sessions**
**REP**	40.60<br>(25.58, 55.61)*<br>*44%***	38.51<br>(21.12, 55.91)<br>*42%*	13.37<br>(5.61, 21.12)<br>14%
**REP+Facilitation**	60.18<br>(34.92, 85.44)<br>51%	41.02<br>(25.77, 56.26)<br>*35%*	15.78<br>(10.78, 21.27)<br>*13%*
**REP+Coaching**	29.32<br>(20.61, 38,03)<br>*44%*	23.70<br>(11.19, 36.20)<br>36%	12.89<br>(7.87, 17.91)<br>*20%*
**REP+Coaching+ Facilitation**	35.60<br>(22.01, 49.19)<br>*48%*	26.21<br>(15.12, 37.30)<br>*36%*	11.94<br>(8.54, 15.33)<br>*16%*

*95% confidence intervals in parentheses. **Percent of Total CBT Delivery.
The cell with the largest effect size is shaded. See Table 7 in main narrative for comparison

Table D4. Pairwise Comparisons for CBT Delivery, by Type (Secondary Outcomes), Using Incomplete Data

Pairwise Comparison of Strategies	Individual Brief Sessions (<15 minutes)	Individual Full Sessions (15+ minutes)	Group Sessions
Secondary Aim Comparisons			
**REP vs. REP+Coaching+Facilitation**	5.00<br>(-14.30, 24.29)<br>*0.07**	12.30<br>(-7.94, 32.54)<br>*0.21*	1.43<br>(-6.33, 9.19)<br>*0.06*
**REP vs. REP+Facilitation**	-19.58<br>(-47.46, 8.29)<br>*-0.27*	-2.51<br>(-23.52, 18.50)<br>*-0.04*	-2.41<br>(-10.04, 5.22)<br>*-0.09*
**REP vs. REP+Coaching**	11.28<br>(-5.11, 27.66)<br>*0.15*	14.81<br>(-6.63, 36.26)<br>*0.25*	0.48<br>(-8.57, 9.53)<br>*0.02*
**REP+Facilitation vs. REP+Coaching**	30.86<br>(5.03, 56.69)<br>*0.42***	17.32<br>(-1.42, 36.06)<br>*0.29*	2.89<br>(-4.40, 10.17)<br>*0.11*
**REP+Facilitation vs. REP+Coaching+Facilitation**	24.58<br>(-3.70, 52.85)<br>*0.34*	14.80<br>(-4.27, 33.88)<br>*0.25*	3.84<br>(-2.98, 10.66)<br>*0.15*
**REP+Coaching vs. REP+Coaching+Facilitation**	-6.28<br>(-19.55, 6.99)<br>*-0.09*	-2.52<br>(-17.29, 12.26)<br>*-0.04*	0.95<br>(-4.09, 5.99)<br>*0.04*

* Effect sizes were calculated based on the following estimated standard deviations: 73.0 for Individual Brief Sessions, 59.9 for Individual Full Sessions, 25.6 for Group Sessions). **The cell with the largest effect size is shaded. See Table 8 in main narrative for comparison.

References

[1] S. M. Shortreed, E. Laber, T. S. Stroup, J. Pineau, and S. A. Murphy, "A multiple imputation strategy for sequential multiple assignment randomized trials," *Stat. Med.*, vol. 33, no. 24, pp. 4202–4214, Oct. 2014, doi: 10.1002/sim.6223.

[2] D.B. Rubin, "Multiple Imputation for Nonresponse in Surveys," in *Multiple Imputation for Nonresponse in Surveys*, John Wiley & Sons, Ltd, 1987, pp. i–xxix. doi: 10.1002/9780470316696.fmatter.

[3] J. Schafer, "Multiple imputation: a primer," Feb. 01, 1999. https://journals.sagepub.com/doi/10.1177/096228029900800102?url_ver=Z39.88-2003&rfr_id=ori:rid:crossref.org&rfr_dat=cr_pub%20%200pubmed (accessed Oct. 12, 2021).

[4] StataCorp, *Stata Statistical Software: Release 16*. College Station, TX: StataCorp LLC., 2019.

[5] StataCorp, *Stata Multiple-Imputation Reference Manual, Release 16*. College Station, TX: StataCorp LLC., 2019.

[6] "Multiple Imputation in Stata." https://stats.idre.ucla.edu/stata/seminars/mi_in_stata_pt1_new/ (accessed Nov. 15, 2021).
